# Supplementary material for: Cellular velocity, electrical persistence and sensing in developed and vegetative cells during electrotaxis
Source: PLoS One. 2020 Sep 18;15(9):e0239379. doi: 10.1371/journal.pone.0239379 (PMC7500600; doi:10.1371/journal.pone.0239379)
Supplement: S1 File — (PDF) [file pone.0239379.s001.pdf]

Supporting Information

Cellular velocity, electrical persistence and sensing in developed and vegetative cells during electrotaxis

Isabella Guido, Douglas Diehl, Nora Aleida Olszok, Eberhard Bodenschatz,

Max-Planck Institute for Dynamics and Self-organization, Göttingen, Germany

SI Movies

SI Movie1: Electrotaxis of wild type AX2 Dd cells. The cells starved 5 hours with cAMP pulses. The images were acquired every 20 s for 2 hours and the polarity of the electric field was reversed after 60 minutes.
SI Movie2: Visualization of F-actin distribution under the influence of the electric field. AX2 Dd cells starved for 5 hours with cAMP pulses. The images were acquired every 10 s for 10 minutes and the polarity of the electric field was reversed after 5 min.

SI Figures

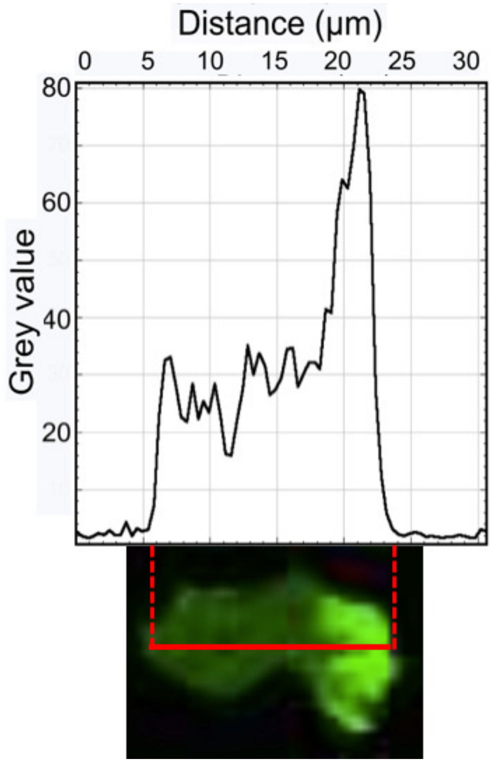

Fig S1. Actin intensity. A line is drawn through the cell and the intensity profile of the pixels along that line is displayed by using the analytical tool of ImageJ.

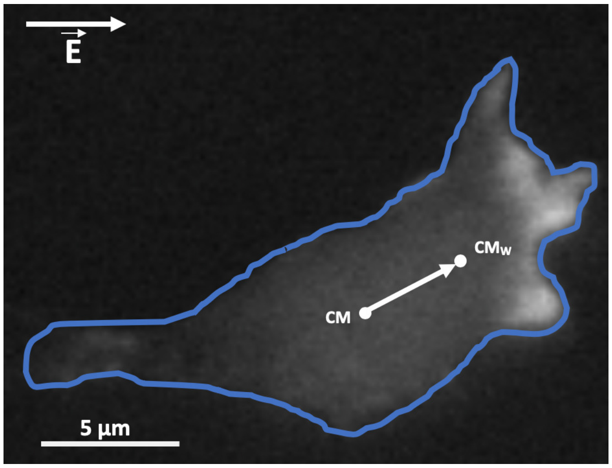

Fig S2. Weigthed center of mass detection. The center of mass CM and the intensity-weighted center of mass CM_w are computed considering the intensity of the pixels representing the actin localization.

Amib_ cell line

A study has demonstrated that amiB is essential for the starvation-induced early-state gene transcription [1]. Interestingly, the most remarkable phenotype of amiB- is an undetectable low level of ACA expression upon starvation and a consequent low level of cAMP relay response. It has been discussed that a major role of amiB is the activation of starvation-induced ACA expression pathways during Dd development. ACA- and amiB- mutant cells demonstrate the same defective cAMP relay and similar electrotactic behaviour without velocity increase over time. That in mind, our experiments allow the conclusion that the intracellular cAMP production machinery plays an important role for the cellular speeding up presented in this study.

[1] Kon T1, Adachi H, Sutoh K., amiB, a novel gene required for the growth/differentiation transition in Dictyostelium. Genes Cells. 2000 Jan; 5(1):43-55

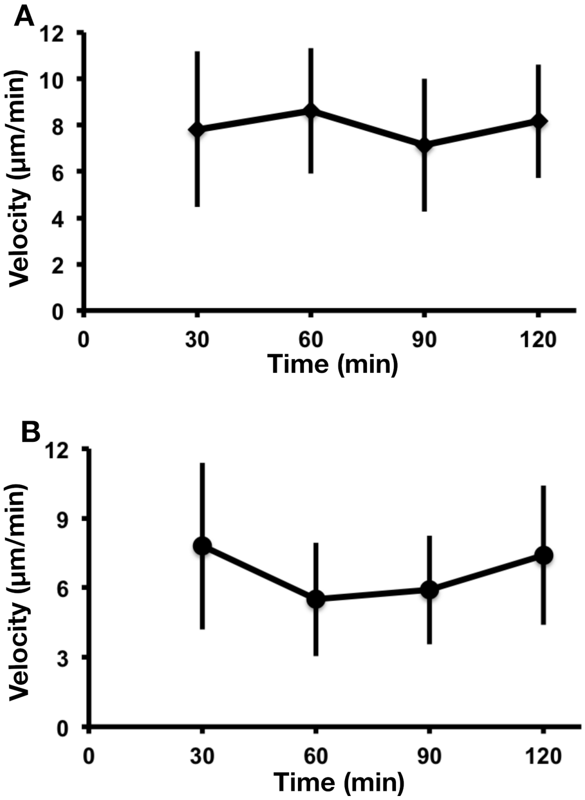

Fig S3. Migration velocity of mutant strains. Migration velocity of mutant strains (A) ACA- and (B) amiB- in an electric field. In both cases the cells migrated towards the cathode with a constant velocity without exhibiting any acceleration as observed in wild type cells. In our opinion this different behaviour could be explained by considering the involvement of transcriptional gene activation required for cell aggregation triggered by nutrient depletion. At the early stage of the differentiation program proteins, essential for production and sensing the cAMP signal, include an adenylyl cyclase (ACA). ACA produces cAMP, which after secretion stimulates nearby cells. Upon this stimulus the cells migrate in direction of the higher cAMP gradient until an aggregate is formed. Therefore, ACA is a key enzyme for establishing this cAMP signalling. Interestingly, ACA- mutants missing the internal production of cAMP do not present any speeding up over time in an electric field typical for wild type cells. By repeating the same experiments with amiB- , a cell strain that is non aggregation-competent, we observed that these cells do not experience an acceleration as well and their velocity stays constant over time.

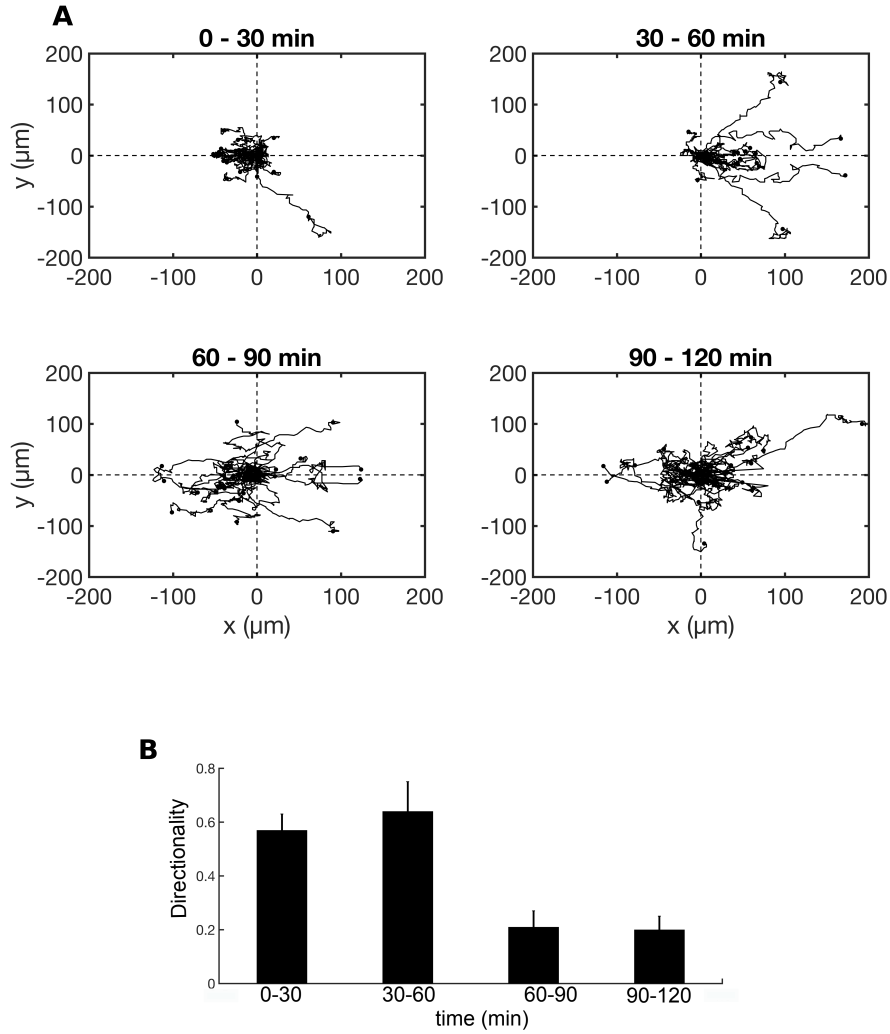

Fig S4. Exemplary cell tracking and directionality. Exemplary cell tracking (A) and directionality (B) of briefly starved cells pulsed with cAMP for 1 hour. A. The cell tracking clearly shows the electrotactic movement in the first hour and the random movement for the rest of the observation time. B. The directionality diagram of the cells shows a sustained cellular migration towards the cathode in the first hour. In the last two time intervals it drops off, indicating the inability of cells to follow the electric stimulus.
